# Trapping and Driving Individual Charged Micro-particles in Fluid with an Electrostatic Device

**DOI:** 10.1007/s40820-016-0087-3

**Published:** 2016-03-10

**Authors:** Jingjing Xu, Zijing Lei, Jingkun Guo, Jie Huang, Wei Wang, Uta Reibetanz, Shengyong Xu

**Affiliations:** 1 Key Laboratory for the Physics & Chemistry of Nanodevices, and Department of Electronics, Peking University, Beijing, 100871 People’s Republic of China; 2 Institute of Microelectronics, Peking University, Beijing, 100871 People’s Republic of China; 3 Medical Faculty, Institute for Medical Physics and Biophysics, University of Leipzig, 04103 Leipzig, Germany

**Keywords:** Electrostatic tweezers, Charged particles, Coulomb potential well, Manipulation, Trap

## Abstract

A variety of micro-tweezers techniques, such as optical tweezers, magnetic tweezers, and dielectrophoresis technique, have been applied intensively in precise characterization of micro/nanoparticles and bio-molecules. They have contributed remarkably in better understanding of working mechanisms of individual sub-cell organelles, proteins, and DNA. In this paper, we present a controllable electrostatic device embedded in a microchannel, which is capable of driving, trapping, and releasing charged micro-particles suspended in microfluid, demonstrating the basic concepts of electrostatic tweezers. Such a device is scalable to smaller size and offers an alternative to currently used micro-tweezers for application in sorting, selecting, manipulating, and analyzing individual micro/nanoparticles. Furthermore, the system offers the potential in being combined with dielectrophoresis and other techniques to create hybrid micro-manipulation systems.

**Electronic supplementary material:**

The online version of this article (doi:10.1007/s40820-016-0087-3) contains supplementary material, which is available to authorized users.

## Introduction

In the past two decades, characterization and application of micro/nanoparticles have attracted much attention. In this field, micro-tweezers have played an important role in capturing and manipulating micro/nanoscale particles, molecules, and atoms. For instance, optical tweezers are using one or multiple laser beams to create a gradient field towards the center of the laser focal point, offering a unique way to trap micro/nanoparticles. Furthermore, it has been shown that atoms can be trapped and cooled in studies on Bose–Einstein condensation [1–3], atomic clocks, and quantum phenomena [4–7]. Optical tweezers have also been extensively applied in a variety of studies on micro-particles [8–10], cells [10], bacteria [11], sub-cell organelles, bio-macromolecules [12, 13], and nanoparticles [14, 15]. Another kind of effective tweezers based on dielectrophoresis (DEP) technique has been applied in sorting [16–19], trapping [20], characterizing [17], and transporting [20] dielectric particles, cells, and bio-macromolecules by polarizing and driving them in non-uniform electric fields [21, 22]. The “Paul Trap,” which utilizes high-frequency alternating electric fields on four specially designed electrodes, is capable of capturing charged particles [23, 24]. Magnetic tweezers, which utilize gradient magnetic fields as the trapping force, can shift and rotate a magnetic particle or other targeted objects modified with a magnetic bead [25–29]. This type of tweezers has been successfully applied in studying the role of topoisomerase in unwinding of DNA [30]. The concepts of electron tweezers which utilize a focused electron beam in a transmission electron microscope (TEM) for trapping and manipulating quantum dots and nanoparticles have also been addressed [31–33]. In addition, optofluidic systems are getting more and more attention in the field. Kayani et al. summarized not only the manipulation forces in controlling particles, but also the applications of optofluidics incorporating controlled particles in manipulating, sensing, and analyzing micro-particles and even bio-molecules nowadays and in the future [34]. There are also other related techniques applied in complex biosensing [35] and biological measurements [36]. All manipulation technologies mentioned above operate mainly on the basis of electromagnetic interaction. In fact, at the micro/nanoscales, the electromagnetic interaction is the only force work effectively among the known four fundamental forces in physics.

In live biosystems, a huge number of effective, rapid, and regular interactions occur every second among intracellular micro/nanoparticles and macromolecules, such as handling, transfer, specific binding, and catalytic reaction [37–43]. Logically, it is convincing that certain ordered manipulation and interaction mechanisms, rather than thermal motion alone, exist in these intracellular activities. However, in live biosystems, there is no essential condition for artificial optical tweezers, magnetic tweezers, DEP, or “Paul traps,” which all need additional power supplies. Computational simulations have shown that the specific interaction between charged particles and local electric field contributed by charge distribution plays the key role in the complex but effective biological processes [44–51]. This is consistent with experimental evidences, which demonstrate that molecules (such as enzymes, DNA, proteins, etc.) in cells usually carry net charges, or even a specific charge distribution [50–56]. These facts lead to a new concept of tweezers, the electrostatic tweezers (EST), which work passively and do not require manmade powers, therefore they could exist in a living biosystem. In a study on the interaction between sunitinib and its specific target protein, Malinska et al. have found that the electrostatic interaction is a major factor, and sunitinib can adjust its conformation to fit the binding pocket as to enhance the electrostatic interactions [57].

As bio-macromolecules are in nanoscale, it is hard to observe the working status of the proposed natural ESTs or traps in a living cell directly. The first report on a nanosized electrostatic trap was reported in a TEM study of cadmium selenide (CdSe) quantum dots, where a CdSe nanocrystal was observed floating and rotating over a carbon film [31]. In this work, the concept and feasible constructions of one-dimensional (1D), 2D, and 3D electrostatic traps were systematically discussed. The first manmade electrostatic trap was reported by Krishnan and co-workers [58]. They have found that by utilizing net electrostatic charges unintentionally left in a nanoscale slit of a microfluidic channel, which resulted in a Coulomb potential, the device was capable of trapping gold nanoparticles and polymer beads. However, this kind of trap cannot capture particles selectively in a controllable way, or release them when needed. To the best of our knowledge, practical electrostatic manipulation devices with functions of driving, trapping, and releasing charged particles have not been reported.

In the present work, we attempted to demonstrate the basic concepts of EST: capture, manipulation, and release of charged particles using a controllable static Coulomb potential well. The electrostatic potential traps used in this work were formed by electrostatic charges distributed on specially designed metallic electrodes. Instead of using naturally formed electrostatic charges, the surface charge density was adjusted with a small external DC voltage source, making the resulting Coulomb force controllable. The limited height of the microfluidic channel of our devices supported the Coulomb potential wells in firmly trapping charged micro-particles. Additional electrodes were also used to apply an external electric field for driving and manipulating particles in the fluidic channels. Recorded with a video camera, motions of confined particles and clusters inside the Coulomb wells showed that these electrostatic devices work well as designed.

## Experimental

### Simulation of the Electrostatic Coulomb Potential Trap

**Fig. 1:**
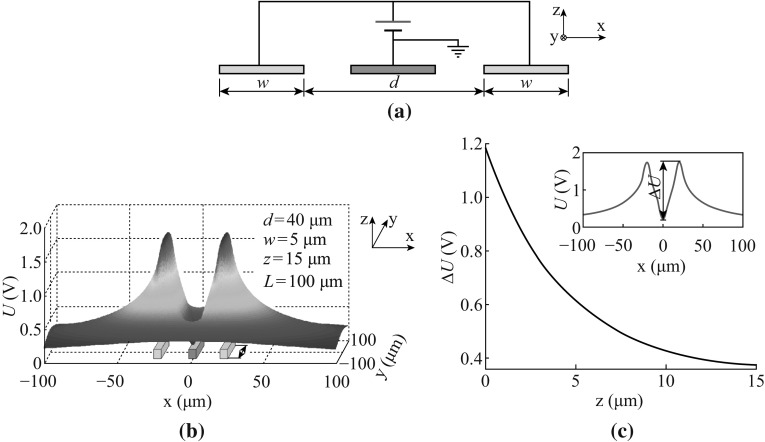
Design and simulation of a 3-stripe electrostatic trap. **a** The schematic structure of the 3-stripe metal electrodes, viewing in *y* direction. **b** Simulation result of electrostatic Coulomb potential in vacuum in the *x*–*y* plane for 3-stripe trap at an excitation voltage of 2 V, at *z* = 15 μm. **c** The curve of the potential depth Δ*U* at *z* direction due to screen effect. The *inset* at the up right corner defines the potential depth Δ*U*

Basically, when a metallic thin-film strip is connected to a static voltage power supplier, its surface will be covered with a certain amount of static charges so as to maintain a constant potential. A prototype device with three parallel metallic stripe electrodes was designed to show the basic features of an electrostatic Coulomb well. Figure 1 illustrates the Coulomb potential in an *x*–*y* plane and at a constant distance to the 3-stripe electrodes. Figure 1a shows the schematic structure of the 3-stripe electrodes, viewing in *y* direction, where the electrodes have a width of *w* and a length of *L*, and the spacing for the outer two electrodes is *d*. The definition for *x*, *y*, and *z* coordinates is presented at the upper right corner. With suitable choices of the voltages applied to the stripes, and at suitable distance of *z*, the deep potential well is formed in the *x*–*y* plane.

Figure 1b plots a typical electrostatic Coulomb potential distribution in vacuum in *x*–*y* plane at *z* = 15 μm, for a 3-stripe trap with *w* = 10 μm, *d* = 50 μm and *L* = 100 μm, where a voltage of 2 V is applied between the positively outer stripes and the earthed central stripe. The simulation of the Coulomb well is accomplished by MATLAB 2012b, and here successive over-relaxation method (SOR) is applicative to solve Laplace equation:

$$\frac{{\mathop \partial \nolimits^{2} \mathop U\nolimits^{{}} }}{{\mathop \partial \nolimits^{{}} \mathop x\nolimits^{2} }} = \frac{{\mathop \partial \nolimits^{2} \mathop U\nolimits^{2} }}{{\mathop \partial \nolimits^{{}} \mathop y\nolimits^{2} }} = \frac{{\mathop \partial \nolimits^{{}} \mathop U\nolimits^{{}} }}{{\mathop \partial \nolimits^{{}} \mathop z\nolimits^{2} }} = \mathop 0\nolimits_{{}}$$

with the boundary conditions that *U* = 2 V on the outer two electrodes, while *U* = 0 on the middle electrode and in the infinite. Considering the width (*w*) and length (*L*) of the electrode, the boundary conditions are expressed in the following form, respectively:

$$U\left( { - \left( {\frac{1}{2}d + w} \right) < x < - \frac{1}{2}d,\quad - \frac{1}{2}L < y < \frac{1}{2}L,\quad z = 0} \right) = U\left( {\frac{1}{2}d < x < \frac{1}{2}d + w,\quad - \frac{1}{2}L < y < \frac{1}{2}L,\quad z = 0} \right) = 2$$

$$U\left( { - \frac{1}{2}w < x < \frac{1}{2}w,\quad - \frac{1}{2}L < y < \frac{1}{2}L,\quad z = 0} \right) = 0$$

$$\mathop U\nolimits_{\infty } = \mathop 0\nolimits_{{}}.$$

In fact, the simulation results in Fig. 1 are based on simplified models, therefore they are qualitative analysis, and give a visible picture for the potential well used in this work. The situation for a real device under test is much more complicated. We have discussed some of the effects in Sect. 4, such as Brownian motion, screen effect, electroosmosis, and thermophoresis.

Such a trap is suitable for capturing positively charged objects. Generally, the depth of the trap, either in the potential (*U*) or in energy (i.e. *qU*, assuming that the charged particle has a charge *q*), decreases rapidly with increasing height (in *z* direction). When the screen effect is taken into account, the trap depth becomes even smaller than the stimulated values (Fig. 1b) in vacuum, because the screen effect remarkably reduces the effective surface charge density at the trap electrodes. It is stimulated by COMSOL 4.3b that the potential depth Δ*U* at *z* values (Fig. 1a) of 0, 5, 10, and 15 μm are 1.18, 0.61, 0.43, and 0.38 V, respectively, as seen in Fig. 1c. Nevertheless, the results mentioned later confirm that the remaining depth of the traps is still deep enough to trap individual or cluster of the charged micro-particles. In our device, as the medium is de-ionized (D.I.) water, the Debye length is in the order of 1 μm, which is deduced from the equation: *λ*_D_ = (8π*l*_B_ *c*_∞_)^−1/2^. Here *l*_B_ is Bjerrum length in water (0.71 nm for univalent ion) and *c*_∞_ is salt ion concentration (10^−7^ M in D.I. water).

### Device Fabrication

**Fig. 2:**
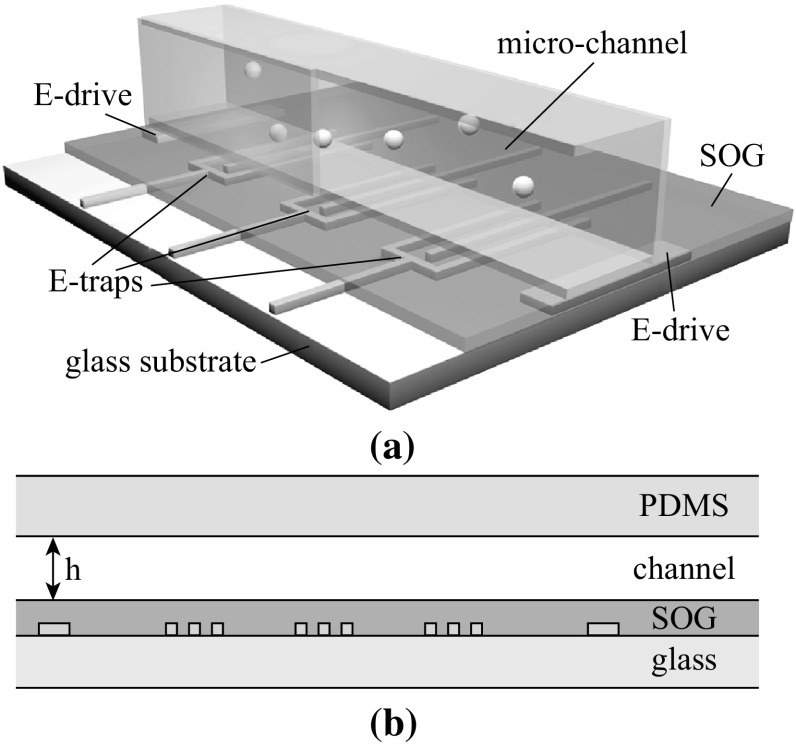
Illustration of the structure and materials for the electrostatic micro-tweezers. **a** A 3D view of the electrostatic micro-tweezers in a microfluid, which includes driving electrodes, trapping electrodes, and the microfluidic channel. **b** Front view of the device showing the structure and materials

**Fig. 3:**
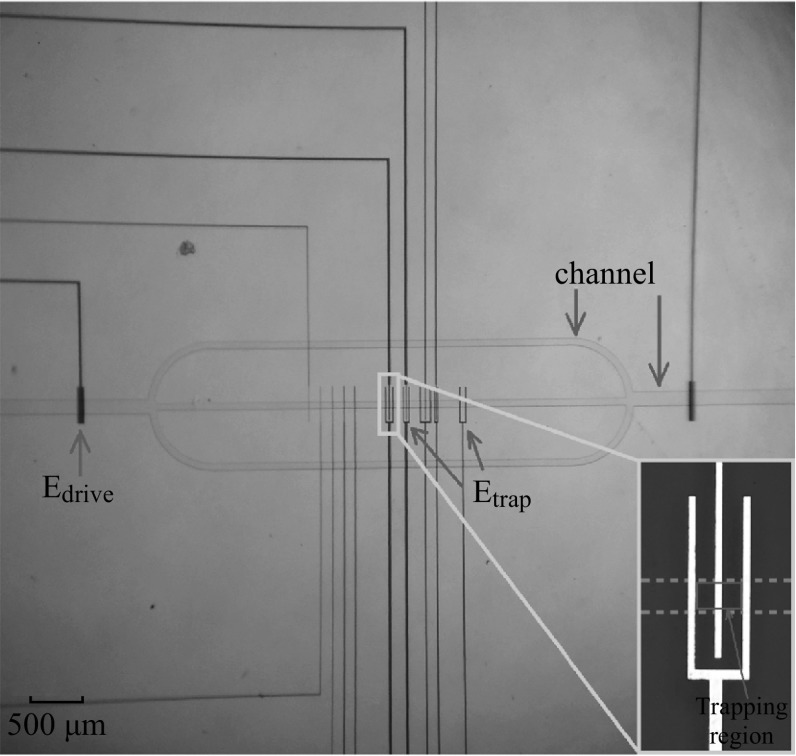
Photograph of the electrostatic micro-tweezers. The *inset* shows the enlarged images of the trap, where the location of the fluidic channel is highlighted with *dashed green lines*. (Color figure online)

Figure 2a is a 3D illustration of the whole device. The whole device was fabricated on a piece of glass substrate. The metallic stripes of the electrostatic traps and the channel are separated by a dielectric layer (SOG in the illustration). Figure 2b is a front view of Fig. 2a through the channel, showing the structure and materials of the device. Note that our traps are not truly 3D. However, the limited height *h* of the fluidic channel sets the maximum *z* (see definition in Fig. 1) for micro-particles moving inside the channel and therefore allows the electrostatic trap to work properly. Figure 3 is an optical photograph of an actual fabricated device in vertical view.

A set of driving electrodes, marked as *E*-drive in Figs. 2 and 3, offers an electric field in the desired direction to drive target particles in the microchannel. When the target particles move into the trapping region (the region between two outer parallel metallic electrodes in one electrostatic trap, marked by the blue box in the inset of Fig. 3), an external static voltage is supplied to the electrode stripes, which activates the trap and keeps the particles captured. Turning off of the external static voltage deactivates the trap and the target particles will be driven out of the trapping region quickly under the driving field.

The electrode substrates were fabricated with standard micro-electro-mechanical system (MEMS) techniques in a clean room. Briefly, as schematically shown in Fig. 2b, a Ti/Au film (5/100 nm) was patterned on a 4″ glass wafer by photolithography (Instrument: SUSS, MBJ4), coating process (Instrument: Kurt J. Lesker, PVD 75), and lift-off process to function as the electrodes [59]. Then, a 300-nm-thick spin-on-glass (SOG) (Futurrex, Inc, China) was spin-coated on the wafer as the dielectric layer. The fluidic channel was prepared by traditional polydimethylsiloxane (PDMS)-based soft lithography approach. PDMS (DOW CORNING, China) pre-polymer (curing agent: base = 1:10) was poured onto a micro-fabricated master (silicon wafer etched by deep reaction ion etching, DRIE) with designed feature sizes, and cured at 70 °C for 1 h. Afterwards, the PDMS was peeled off from the master and holes were punched to form the inlet/outlet. Finally, the PDMS microchannel was bonded with the electrode substrate after oxygen plasma treatment for 50 s. After the microscale processing, the device was bonded to a Printed Circuit Board for convenient manipulation and measurement.

Microscope (NOVEL) and Color Video Camera (JVC:TK-C9211EC) were used for observing the motion of micro-particles in the fluidic channels and for recording videos. As shown in Fig. 1, the microscope observation was taken along the *z* direction, i.e., for motion of micro-particles in a 3D space (in the fluidic channel), and all the recorded images and videos were indeed projections of the motion in the *x*–*y* plane. In order to identify the transparent polystyrene (PS) particles in the microchannel, the asymmetric light beam from microscope was irradiated on the channel.

### Surface Charge Density of Micro-particles

**Fig. 4:**
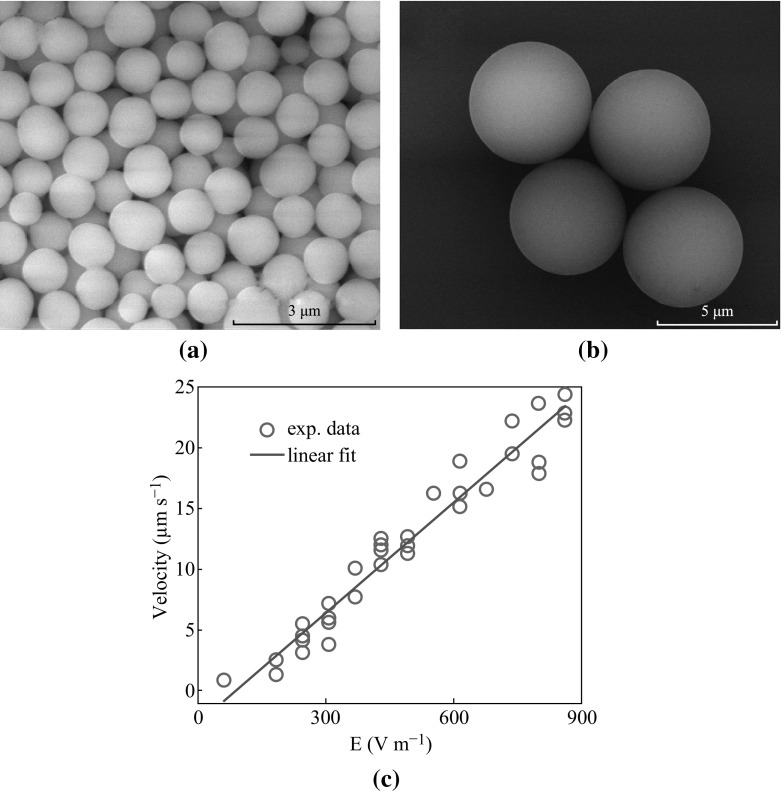
Characterization of charged micro-particles used in the experiments. **a**, **b** SEM micrographs of SiO_2_ and polystyrene particles, showing an average diameter around 1 μm and near 5 μm, respectively. **c** The *red circles* are measurement results of the velocity versus applied driving electric field in the fluid for 33 individual 5-μm-diameter PS particles. The *blue line* is a linear fit. (Color figure online)

Two kinds of micro-particles have been applied in the experiments, namely 1-μm-diameter negatively charged SiO_2_ particles (Baseline, China) and 5-μm-diameter polystyrene (PS) particles (Baseline, China) which exhibit positive charge due to surface-bound amino groups. Figure 4a, b shows their scanning electron microscopy (SEM) micrographs, respectively.

In a first step, the surface charge densities of these micro-particles were determined. The particles were suspended in D.I. water and their motion under directional driving electrostatic fields from zero up to 1000 V m^−1^ has been observed. The velocity of each individual micro-particle was measured directly by comparing its position in the video frames taken at different points in time. Figure 4c plots measurement data from 33 PS micro-particles. A linear fit of the data gives a gradient ~3 × 10^−8^ m^2^ (V s)^−1^. Note that the fit line does not tend to zero due to the existence of viscous drag, which delays the movements of the particles until the driving force is large enough. After achieving equilibrium, the motion of the micro-particles follows the Stokes Law, *f* = 6π*rηv*, where *f* is the viscous drag (≈driving force at equilibrium), *r* the radius of the particle, *η* the fluid viscosity, and *v* the particle velocity. Here the driving force (Coulomb force) *f* approximately fulfills *f* = *qE* = *qU*/*d*, where *d* and *U* are the distance between two electrodes and the applied voltage, respectively. From the two equations above, taking *η* as 890 μPa s, an average surface charge density of 1.1 × 10^−4^ *e* nm^−2^ is derived for the 5-μm-diameter PS particles (where *e* is the elementary charge with a value of ~1.6 × 10^−19^ C). Similarly, the average surface charge density for the 1-μm-diameter SiO_2_ micro-particles was calculated to be 2.7 × 10^−4^ *e* nm^−2^ (data not shown). It has to be mentioned that the surface charge density of the micro-particles studied in this work is about 3–4 orders of magnitude lower than that observed in some bio-macromolecules.

The SiO_2_ micro-particles do exhibit a ~2.5 times higher net charge density than the PS particles. As the surface area of the PS particles is ~25 times larger, each PS particle roughly carries 10 times more charges than an SiO_2_ particle. Thus, a total particle charge of ~7900 *e* (PS) and ~−830 *e* (SiO_2_) can be calculated.

## Results

### Driving Micro-particles in a Microchannel

**Fig. 5:**
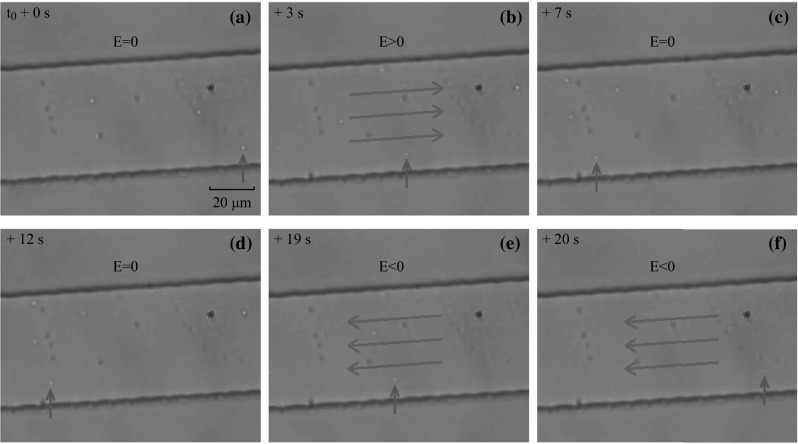
Video frames showing device’s manipulation for driving negatively charged SiO_2_ directly. **a**–**c** The particle indicated by a *blue arrow* is driven from the right side to the left when applying electric field towards right. **c**, **d** The particle stops and takes Brownian Motion when turning off the applied voltage. **d**–**f** The particle moves backwards when changing the direction of applied electric field, and the velocity increases with the driving voltage. (Color figure online)

With the help of an external field, charged micro-particles could be driven inside the microchannels, as shown in Fig. 5 (see Video 1 in supplemental file). Here negatively charged SiO_2_ particles, appearing as bright white dots in the video, do not show any direct movement when no external field is applied, as shown in Fig. 5a. Once a DC voltage is applied with the field direction from left to right (highlighted with red arrows), the negatively charged SiO_2_ particles start to move from right to left in the microchannel as shown in Fig. 5a–c. Upon reversing the direction of the driving field, the particles then move backwards from left to right, as shown in Fig. 5d–f. It has to be mentioned that the moving velocity of these particles can be controlled by the driving field intensity. After the driving field is switched off, the particles return to a stationary status with observable Brownian motion.

### Capture of Individual Micro-particles

**Fig. 6:**
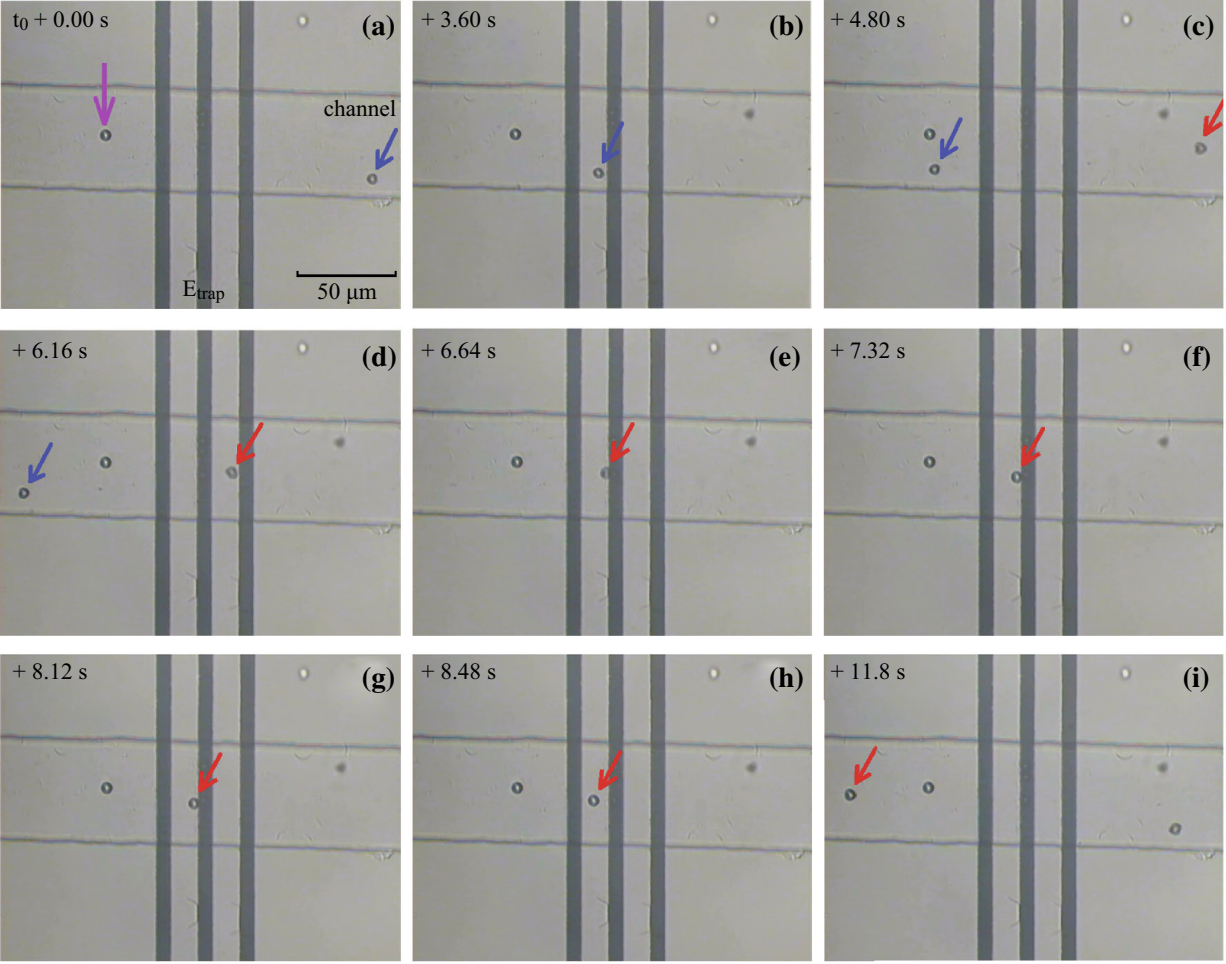
Video frames showing capture of a single PS particle with a 3-stripe electrostatic trap. The nine photo images are taken from a movie, which records the functions of trapping and releasing individual PS particles, which are positively charged. A constant driving electric field is applied in parallel to the microchannel from right to left. The number marked on the *upper left corner* of each frame indicates the time when the frame is taken as compared to the first one. **a**–**c** The trap is not excited, so the particle marked with a *blue arrow* moves freely across the trapping region. **d**–**g** The trap is turned on, and a PS particle marked with a *red arrow* is trapped near the central electrode. **h**, **i** The trap is turned off, thus the trapped particle quickly leaves the trapping region. (Color figure online)

**Fig. 7:**
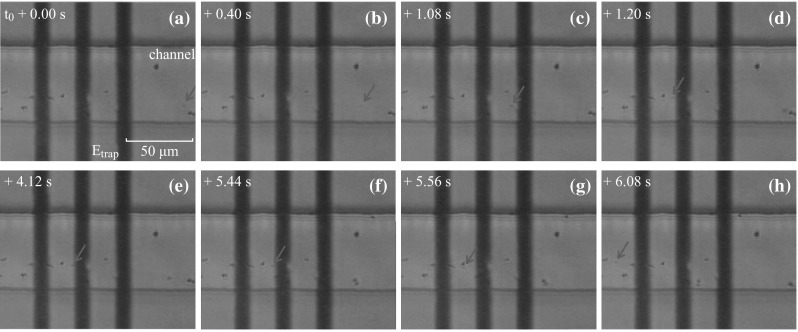
Video frames showing trapping and releasing operation for an individual SiO_2_ micro-particle (marked with a *red arrow*) with a 3-stripe electrostatic trap. (Color figure online)

Capture of individual charged micro-particles in a controllable way is the key function of electrostatic traps. Suspensions of individual charged PS and SiO_2_ particles were used to verify the controllability and effectiveness of the device, as shown in Figs. 6 and 7.

Figure 6 shows a set of video frames (see Video 2 in supplemental file) demonstrating the real-time observation of PS micro-particles in a 3-stripe trapping device. As the original video has a sampling speed of 25 frames per second, the position of individual PS particles at a certain point in time could be determined. The starting time of the first frame is set as *t*_0_, and the time points for consequent frames are determined by the number of frames in between, utilizing a constant time interval of 40 ms between adjacent frames. This time point is then highlighted on the upper left corner of each frame.

In Fig. 6a, the particle marked with a purple arrow is a PS micro-particle firmly adsorbed on the wall of the channel, and therefore does not move in the whole observation period. The time *t*_0_ is chosen when a moving PS particle (highlighted with a blue arrow) occurs in the view of the CCD video camera. No excitation voltage is applied to the 3-stripe electrostatic trap, so the PS particle passes through the trapping region with a constant velocity (~39 μm s^−1^), under the driving electric field.

At the time *t* = *t*_0_ + 4.80 s, a second PS particle (indicated by a red arrow) appears in the observation region (Fig. 6c), moving at a constant velocity similar to the first one. When the second particle moves into the trapping region, which is roughly situated within the area confined by the two inner edges of the positively charged metallic stripes (Fig. 3), at *t* ≈ *t*_0_ + 6.00 s, a DC voltage of 2 V is applied to the 3 electrodes by manual control, activating the electrostatic potential well. Immediately, the second particle is captured near the central electrode. After a while, the trap is deactivated (*t* ≈ *t*_0_ + 8.00 s), releasing the particle from the trap. Subsequently, the particles started to move under the driving electric field again.

During the period when the second PS particle is trapped (from *t* = *t*_0_ + 6.64 s to *t* = *t*_0_ + 8.12 s), the particle is not fully stopped. Indeed, one sees that it is vibrating and oscillating at the original trapped location.

After reversing the voltage applied on the trap electrodes, the negatively charged SiO_2_ particles can be captured by the same device. This shows the flexibility of such electrically controlled electrostatic trap. Figure 7 shows a series of 8 video frames (see Video 3 in supplemental file), illustrating the motion of 1-μm-diameter SiO_2_ particles inside the microchannel. Here *t*_0_ is again set at the point when an SiO_2_ particle, indicated by the red arrow in Fig. 7a, occurs under a small driving electric field. The particle moves quickly from the right to the left in the observation area. At *t* ≈ *t*_0_ + 1.00 s, the trap is activated and then captures the particle at the central region of the trap (the minimum region of the Coulomb potential well). The particle remains in the trap until the trap is deactivated manually at *t* ≈ *t*_0_ + 5.00 s. Afterwards, it moves out of the trapping region along the original direction, as shown in Fig. 7f–h.

**Fig. 8:**
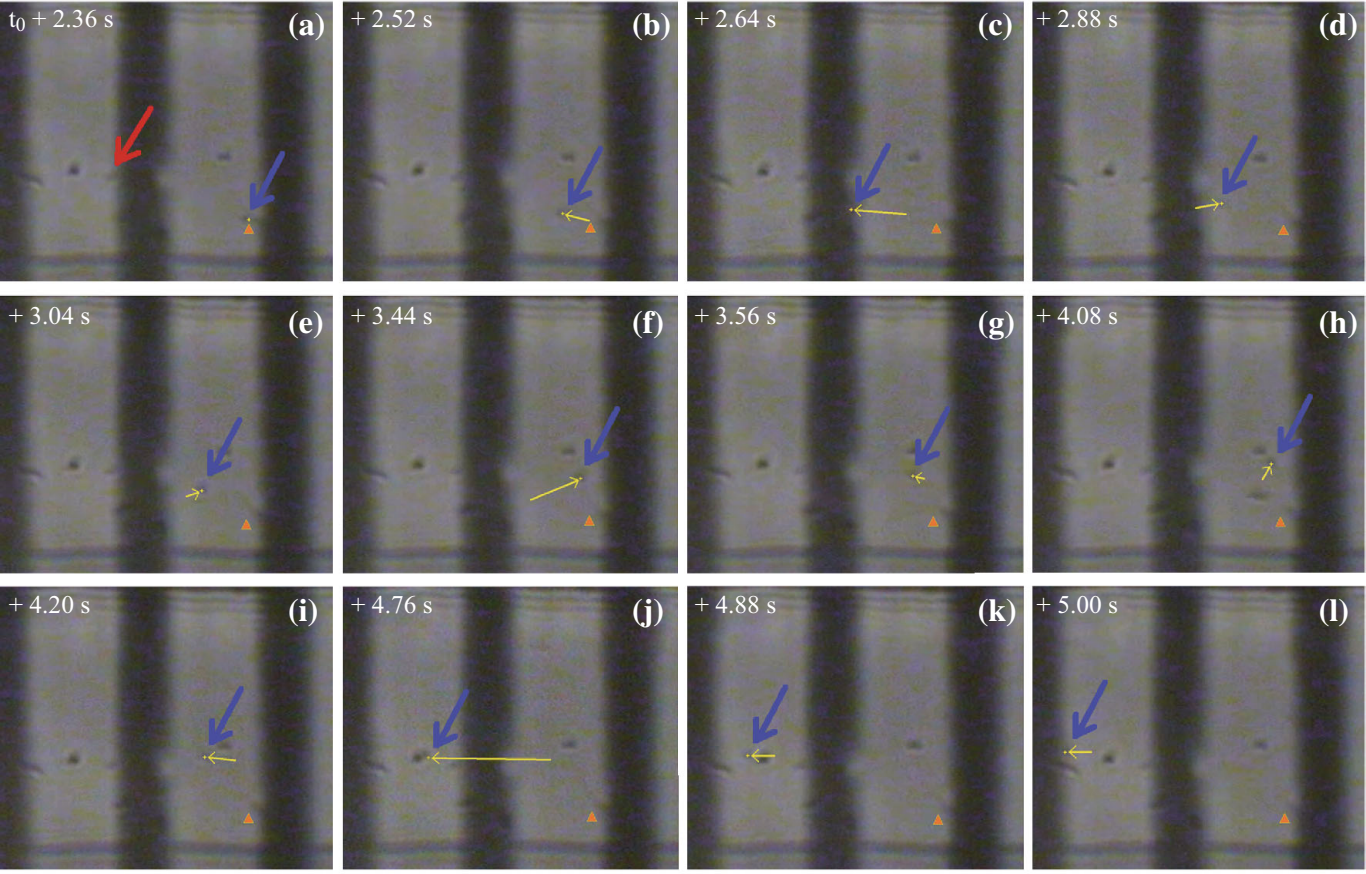
A set of photos showing the motion track of another SiO_2_ particle (marked with a *blue arrow*) while the first particle is kept trapped. The initial location of the second particle is marked with an *orange triangle*, and each move of this particle from its previous position is highlighted with a *yellow line*. (Color figure online)

The 12 frames in Fig. 8 are magnified images of the trapping region shown in Fig. 7, and describe the motion of another SiO_2_ particle, exhibiting an interesting phenomenon. In the period from *t* ≈ *t*_0_ + 2.36 s to *t* ≈ *t*_0_ + 5.00 s when the SiO_2_ particle mentioned in Fig. 7 is kept trapped near the central electrode, another SiO_2_ particle (marked by the blue arrow) is moving inside the trap. It is previously adsorbed to the right electrode before the trap is activated. However, this SiO_2_ particle starts to move at *t* ≈ *t*_0_ + 2.36 s, moving leftwards or rightwards within the trapping region. A small yellow dot is used to highlight the center of this moving particle, and the location of its first appearance in the view region is marked with an orange triangle. For each two successive frames, the shift of the particle location is highlighted with a yellow vector. The behavior of this particle indicates that a sufficient trapping region exists between the two outer electrodes, which is consistent with our simulation results in Fig. 1.

### Capture of Individual Micro-particle Clusters

**Fig. 9:**
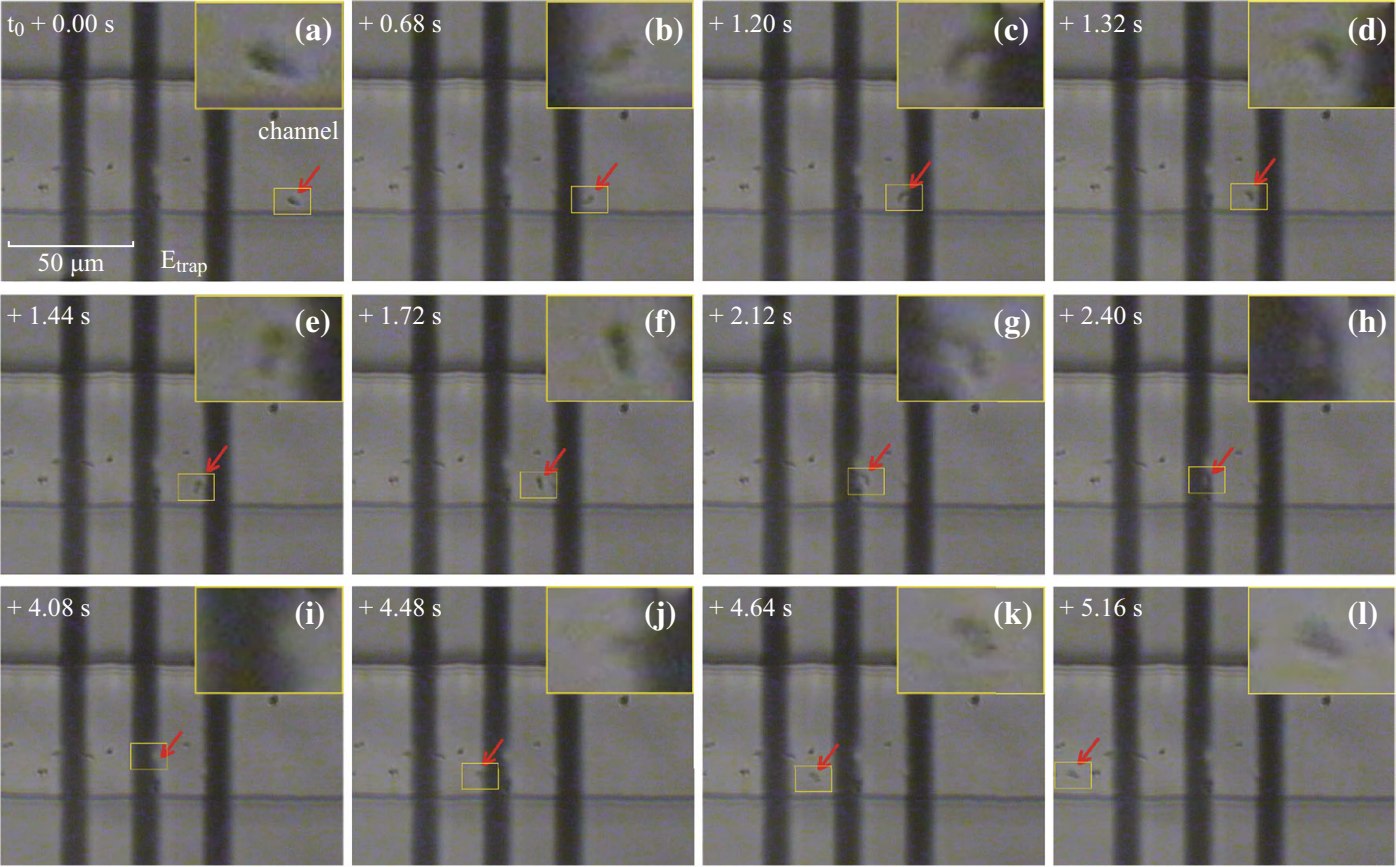
Video frames showing capture of a cluster of SiO_2_ micro-particles with a 3-stripe electrostatic trap. **a**, **b** The trap is in an idle state, and the cluster is moving freely under an electrical driving force. **c**–**j** The trap is turned on, and the cluster is shaking, rotating, and moving slightly in the trap. **k**, **l** The trap is tuned off, and the cluster moves out of the trapping region. The *inset* of each frame is an enlarged image of the cluster in the region marked with a *yellow rectangle*, showing roughly the 3D orientation of the cluster in the fluid. (Color figure online)

The device shows the potential of trapping not only individual micro-particles, but also micro-particle clusters, as shown in Fig. 9. Here a cluster of SiO_2_ particles is naturally formed in the fluidic microchannel, and captured by a 3-stripe electrostatic trap. The cluster has the shape of an asymmetric chain, so when it rotates randomly in the fluid, probably due to Brownian motion, the shape of its projection in the microscopy images (i.e., in the *x*–*y* plane, see definition in Fig. 1) keeps on changing.

The 12 frames displayed in Fig. 9 are taken from a 5.16-s-long video (see Video 4 in supplemental file), whereas *t*_0_ is the time when the micro-particle cluster (indicated by the red arrow) appears in the visual field of the CCD camera. The negatively charged cluster is driven from the right towards the left by a constant electrical field. The electrostatic trap is turned on at *t* = *t*_0_ + 1.20 s, when the cluster just moves into the region of Coulomb potential well. After the trap is turned off at *t* = *t*_0_ + 4.48 s, the cluster starts to move out of the trapping region.

The inset in each frame in Fig. 9 is an enlarged image of the marked yellow region showing the shape of the cluster in detail. One sees that during the period from *t* = *t*_0_ + 1.32 s to *t* = *t*_0_ + 2.40 s, the trapped cluster is still in a status of slow motion. It changes position in the trap and meanwhile rotates randomly. These are the typical features showing the nature of Coulomb potential well designed in this work.

## Discussion

As illustrated in Fig. 1, the Coulomb potential well used in the present device as ESTs has a wide trapping region in the *x*–*y* plane, and a particle being trapped may find local energy minimum locations. Several physical mechanisms of a microfluid under electrical fields, such as Brownian motion, screen effect, electroosmosis, and thermophoresis, may influence the trapping performance. The method described in this work is similar to DEP technique, but still there is an essential difference between these two methods.

### Effect of Brownian Motion

Our simulation results show that, with an excitation voltage of 1 V, the corresponding depth of the Coulomb potential well for a micro-particle carrying a net charge of 1000 *e* is much higher than the kinetic energy (in the order of 0.1 meV). Thermal energy *k*_B_ *T*, which is 26 meV at room temperature, plays an important role when trapping nanosized particles [60, 61]. But for trapping micro-particles in the current work, the thermal energy is the measure from fluctuation of thermal motion of a vast number of molecules, atoms, ions, and clusters in the solution. These fluctuations do not add up to a net force which could induce directional movement of the micro-particles. They only result in Brownian motion, which has an extremely low kinetic energy compared to the trap depth in energy. Therefore, the thermal energy is not a key issue of concern.

### Screen Effect

The screen effect is a common phenomenon in a microfluidic system. It is mainly caused by the adsorption of ions at the liquid–solid interface [62]. The screen effect remarkably reduces the effective surface charge density at the trap and driving electrodes. As a result, the effective field generated by the driving electrodes is reduced, thus the real average charge each micro-particle carries should be higher than what we have calculated. The curve of potential depth versus height in Fig. 1c has taken the screen effect on trap electrodes rather than the screen effect on particles into consideration; the latter could weaken the trap’s constraint on charged particles.

### Effects of Electroosmosis and Thermophoresis

Electroosmosis is another common phenomenon in a microfluid system, which is mainly determined by the *ζ* potential of the electrical double layer (EDL) and the geometric configuration of the electrodes. In our devices, as the spacing of two counterpart electrodes is only 20 μm, when applying a DC voltage of 1–2 V on the trapping electrodes, a large electric field is generated in the microfluidic channel and vortexes may therefore occur in the trapping region [63, 64]. The micro-particles being trapped could receive additional kinetic energy from the electroosmosis flow. Compared to the depth of the Coulomb potential well of the device, this effect induces only vibration, rotation, and drifting of the trapped particles inside the trapping region, as shown in Videos 1–4 in supplemental files. But the effect could not release the particle from the trap as the electroosmosis flow in D.I. water is weak.

Furthermore, a weak electroosmosis current causes little joule heat, thus not inducing a remarkable increase of the local temperature in the fluid [65]. In other words, the thermophoresis effect is also weak and would not severely affect the trapping performance.

### Similarity and Difference Between the Current Method and DEP Technique

The DEP technique also employs local electrodes with desired patterns. In DEP, non-uniform electric fields, usually generated with an AC power [21, 66], are utilized to trap dielectric particles (either charged or not) [67, 68], and the flow of microfluidics is used to drive the particles [66]. DEP has been applied in high-throughput aggregation and sorting of particles or bio-particles with different characteristics including size, dielectric properties, and bioactivity [69]. However, EST may show the merit of trapping and manipulation of individual nano/micro-particles in certain applications. It is feasible to combine both EST and DEP in one system to trap and manipulate target particles or bio-molecules effectively and precisely.

## Conclusion

In summary, we have demonstrated basic concepts of ESTs, i.e., driving, trapping, and releasing charged micro-particles, with trapping and driving electrodes embedded in a microfluidic device. The surface charge density of the trapping electrodes and the status of the traps, as well as the velocity and direction of the micro-particles, are controlled individually with external DC voltages. As the motion of micro-particles in the microchannel was limited by the height of the fluid channel, the local Coulomb potential well formed by a 3-stripe electrostatic trap cannot be fully considered 3D. Nevertheless, the whole device worked well in the trapping performance for positively charged 5-μm-diameter PS particles and negatively charged 1-μm-diameter SiO_2_ particles. The results showed that the devices could drive, trap, and release individual micro-particles and/or clusters, making it a promising technique for analysis of single micrometer-sized subjects. Motions of random vibration, drifting, and rotation of trapped particles (clusters) indicated clear characteristics of electrostatic traps. Other effects including Brownian Motion, screen effect, electroosmosis, and thermophoresis were discussed, and seem to have noncritical influences on the trapping performance.

The performance of the presented devices can be further improved by the addition of sensing of target objects, or by the usage of more sophisticated electrode structures (e.g., a multi-ring configuration). The 3D shape of the Coulomb well can also be adjusted with a precise control of net charge density distribution on the trapping electrodes, e.g., by fixing earth lines in the microchannels. We believe that the working principle of the current device is applicable to smaller devices; therefore, it may lead to a sophisticated technique of “electrostatic nano-tweezers” for sorting, separation, trapping, and manipulation of individual charged nanoparticles, such as proteins and DNA, which are often naturally charged.

## Electronic supplementary material

Below is the link to the electronic supplementary material.

Supplementary material 1 (MPG 4500 kb)

Supplementary material 2 (MPG 2646 kb)

Supplementary material 3 (MPG 1286 kb)

Supplementary material 4 (MPG 889 kb)
